# Zn-based eutectic mixture as anolyte for hybrid redox flow batteries

**DOI:** 10.1038/s41598-018-24059-x

**Published:** 2018-04-10

**Authors:** Yiyu Wang, Zhihui Niu, Qi Zheng, Changkun Zhang, Jing Ye, Gaole Dai, Yu Zhao, Xiaohong Zhang

**Affiliations:** 1 Institute of Functional Nano & Soft Materials (FUNSOM), Jiangsu Key Laboratory for Carbon-Based Functional Materials & Devices, Soochow University, 199 Renai Road, Suzhou Industrial Park, Suzhou, Jiangsu 215123 P. R. China; 2 Testing & Analysis Centre, Soochow University, 199 Renai Road, Suzhou Industrial Park, Suzhou, Jiangsu 215123 P. R. China

## Abstract

Developing greener batteries with new chemistries is a formidable challenge, and a major focus for years to come. Redox flow batteries are receiving increasing research interest for grid-scale electrochemical energy storage owing to their unique architecture. However, challenges still remain by their low energy density as well as corrosive and/or toxic electrolytes. An anolyte based on aprotic Zn deep-eutectic-solvent, which uses low cost, abundant and environmentally benign materials, exhibits a utilizable concentration of Zn^2+^ ca. 1.7 M, resulting in a reversible volumetric capacity of ca. 90 A h·L^−1^. Combined with high efficiencies and relatively low redox potential of −1.12 V vs. Ag/AgCl, such an anolyte provides an alternative way to explore a family of anolytes using new chemistries for rechargeable Zn batteries that meet the criteria for grid-scale electrical energy storage.

## Introduction

Future rechargeable batteries should include alternative chemistries that are inherently environmental-friendly and elementally abundant. For grid-scale electrochemical energy storage, much attention has been paid to the flow-based electrochemical energy storage systems such as redox-flow batteries (RFBs)^1^. RFBs separate the energy storage and power generation by a split cell design that maintains the redox reactions at the surface of the current collectors inside a stack, while stores the electro-active species in externally in circulated flowing liquid electrolytes^2^. Such a unique liquid electrode design offers independent scaling of energy and power, modular construction, and replaceability of defective components for storing energy^3^. However, the energy density of conventional RFBs based on aqueous electrolytes is relatively low, generally limited to ca. 30 W h·L^−1^. The low energy density arises from the low discharge voltage restricted by the water electrolysis window, limited solubility of the active species, and only one-electron transfer per active molecule in most RFB chemistries^4–7^. Alternatively, taking advantage of multi-electron-transfer redox reaction of metals like Zn and Fe, or low redox potential of alkali metals, hybrid RFBs, which uses one solid-state battery electrode such Zn, Al and alkali metals instead of liquid electrolytes, may offer the possibility to achieve higher energy density^8–10^. Since the volumetric capacity of the electrolyte is usually measured as the amount of energy stored per unit volume, which equals to *Nc*_a_*F*/*n*, where *N*, *c*_a_, *F* and *n* are the number of electrons involved in the redox reaction, the concentration of active redox species, the Faraday constant, and the electrolyte volumes contributing to redox reactions, respectively^11^, an improvement in volumetric capacity requires maximization of both the concentration of active redox species and the number of electrons involved in the redox reaction. Besides, increasing the RFB operating voltage has been hampered to date by migration of the redox molecule with sufficiently negative potential to the anode^12^.

It’s therefore necessary to emphasize that the cost and energy density together with material availability and environmental benignity are major criterions needed consideration for grid-scale electrical energy storage applications. Among the developed anode materials for RFBs, Zn is an interesting material because of its availability, negative electrode potential, and two-electron transfer despite that the capacity of Zn-based hybrid redox flow RFBs is limited by the amount of Zn^2+^ deposited in the anode chamber. For aqueous electrolyte, the reaction between Zn and water usually yields undesired hydrogen evolution^13^. This causes a gradual self-corrosion of Zn and lowers the active material utilization^14^. Meanwhile, aprotic electrolytes are beneficial to the cyclability of the Zn electrodes and are also able to suppress the self-corrosion of Zn and eliminate carbonation^15,16^. However, the solubility of Zn salts is much lower in aprotic electrolytes compared with the aqueous counter parts.

Herein, we report a Zn-based, aprotic deep-eutectic-solvent as anolyte potentially applicable to hybrid RFBs. Deep-eutectic-solvents are systems formed from a eutectic mixture of Lewis or Brønsted acids and bases which can contain a variety of anionic and/or cationic species. Compared with other aprotic solvents or ionic liquids used in RFBs, deep-eutectic-solvents are usually biodegradable, and readily available from inexpensive compounds. In this demonstration, ZnCl_2_ and acetamide have been used to form eutectic solvent based on the considerations: i) eutectic solvents usually exhibit a low vapor pressure, relatively wide liquid-range, and nonflammability, as well as small surface tension, which suggest good wettability with electrode^17^; and ii) Zn^2+^/Zn shows a low redox potential of −0.98 V *vs*. Ag^+^/Ag as well as relatively low overpotential upon electroplating/electrostripping processes. Hence, such a Zn-based eutectic solvent that has self-contained electroactive species may serve as an ideal anolyte for the pursuit of greener RFBs with high volumetric capacities.

## Experimental Section

### Synthesis

To prepare Zn eutectic solvent, acetamide (99%, Alfa Aesar) and ZnCl_2_ (anhydrous, 99.99%, Alfa Aesar) with different molar ratio were mixed under constant stirring at 60 °C to accelerate the formation of Zn eutectic solvent until a transparent liquid formed. To decrease viscosity and enhance ionic conductivity, EC/DMC (3/7, v/v, water content < 30 ppm) and bis(trifluoromethane)sulfonamide lithium salt (LiTFSI, anhydrous, 99.95%, Sigma Aldrich) were added into of the as-prepared Zn eutectic solvent. The density of Zn eutectic solvents with ZnCl_2_ molar fraction of 13.7%, 16.7% and19.4% are 2.02, 2.16, and 2.29 g·cm^−3^, respectively. The concentration of the anolytes used for evaluating Zn electroplating/electrostripping efficiency is adjusted by EC/DMC solvent. The ZnCl_2_ concentration in the Zn-based eutectic solvent are 4, 4.25, 4.5, 4.75, 5, 5.25, 5.5, 5.75, 6, 6.25, 6.5, 6.67 M, and the corresponding density is 2.17, 2.22, 2.27, 2.33, 2.38, 2.43, 2.48, 2.54, 2.59, 2.64, 2.70, 2.73 g·cm^−3^, respectively. The concentration of LiTFSI was fixed to 0.6 M in all Zn eutectic mixtures. To prepare [Fe(phen)_3_](BF_4_)_2_, FeSO_4_·7H_2_O (12 mmol, 98%, Alfa Aesar) and 1,10-phenanthroline monohydrate (36 mmol, 97%, Alfa Aesar) were firstly dissolved in 50 mL distilled water under strong magnetic stirring under N_2_ atmosphere, followed by heating up to 70 °C and maintaining at this temperature for 2 hrs. Then, excess NH_4_BF_4_ (40 mmol, 97%, Alfa Aesar) was added into the solution and maintained at 90 °C for another 2 hrs. Afterwards, the precipitation was collected by centrifuging and washed with a small amount of frozen deionized water for several times to remove NH_4_Cl content. The solid was obtained after solvent removal by spin steaming, followed by filtering with silica gel, and washed with acetonitrile. Finally, Further purification was carried out by drying in vacuum to remove the residual water.

### Characterizations

DSC (Perkin-Elmer DSC-7 calorimeter) measurement was carried out by calibrating with indium and flushed with N_2_ and scanning at a rate of 10 °C·min^−1^ from −70 °C to 100 °C using about 10 μL of the Zn eutectic solvent packed in standard aluminum pans. Fourier Transform infrared spectroscopy (FTIR, Perkin-Elmer spectrophotometer) spectra were collected in the frequency range of 4000–400 cm^−1^ at room temperature with a resolution of 4 cm^−1^. The viscosity and conductivity of the Zn eutectic mixture were determined by a rotary viscometer (NDJ-1 Shanghai Yueping Scientific Instrument Corporation Limited, China) and conductivity meter with four channel conductivity electrodes (STARTER 300 C), respectively. Scanning electron microscopy (SEM, Zeiss Supra 55) image showing the surface structure of Zn foil was recorded after washing with pure ethylene carbonate and dimethyl carbonate. Cyclic voltammetry tests were performed on a potentiostat (CHI760E, CH Instruments) with a three-electrode configuration of with glassy carbon as the working and counter electrodes, and silver chloride electrode as the reference electrode. Galvanostatic cycling performance was performed on a potentiostat (BT-2043, Arbin Instruments) with current density of 0.12 mA·cm^−2^ in the potential range of 1–2.3 V and 0.5–2.5 V for Zn eutectic mixture | [Fe(phen)_3_](BF_4_)_2_ and Zn eutectic mixture|LiI cells, respectively. Rotation disk electrode (RDE, ALS RRDE-3A) voltammetry investigation was carried out using glassy-carbon as working electrode, Pt wire as counter electrode and silver chloride electrode as reference electrode. The electrolyte was composed of 2.5 mM [Fe(phen)_3_](BF_4_)_2_ and 0.25 M LiTFSI in ethylene carbonate and dimethyl carbonate (3:7, v-v). The working electrode was rotated at 625, 900, 1225, 1600, 2025, 2500, 3025, 3600 and 4225 rpm, while the voltage was linearly swept from 0.65 to 1.05 V vs. Ag/AgCl at a sweeping rate of 10 mV s^−1^. NMR spectra (Advance 600 MHz Bruker spectrometer) were recorded using CDCl_3_ (or D_6_-DMSO) with tetramethylsilane (TMS) as the internal standard. ^1^H NMR (600 MHz, CDCl_3_, ppm) of 1,10-phenanthroline: δ = 9.18 (dd, ^*3*^*J* = 4.5 Hz, ^*4*^*J* = 1.8 Hz, 2 H), 8.24 (dd, ^*3*^*J* = 7.8 Hz, ^*4*^*J* = 1.8 Hz, 2 H), 7.78 (s, 2 H), 7.62 (dd, ^*3*^*J* = 7.8 Hz, ^*4*^*J* = 4.2 Hz, 2 H); ^1^H NMR (600 MHz, D_6_-DMSO, ppm) of [Fe(phen)_3_](BF_4_)_2_: δ = 8.80 (dd, ^*3*^*J* = 7.8 Hz, ^*4*^*J* = 1.2 Hz, 2 H), 8.40 (s, 2 H), 7.75 (dd, ^*3*^*J* = 8.1 Hz, ^*4*^*J* = 5.1 Hz, 2 H), 7.71 (dd, ^*3*^*J* = 5.4 Hz, ^*4*^*J* = 1.2 Hz, 2 H) (Fig. S1, Supplementary material).

### Calculation details

The first principles calculations are performed using the Gaussian 09 software based on DFT combined with multiwfn software^18,19^. The structure optimization of [Fe(phen)_3_]^2+^ and [Fe(phen)_3_]^3+^ is performed using the B3lyp as the functional and 6-31 g(d,p)/lanl2dz as the mixed basis sets for C, N, H and Fe, respectively^20^. The structure optimization of Zn (II) complexes is performed using the B3lyp/def2tzvp level of theory^21^. The B3lyp/def2tzvp level of theory was selected to optimize the structure of Zn (II) complexes without imaginary frequencies. The hydrogen bond interaction energy was calculated using B3lyp/6-311 g*. The interaction energy (Δ*E*) between different molecules was calculated as the difference between the total energy of the complex and the individual energy according to Δ*E* = *E*_total_ − *E*_act_ − *E*_x_^22^, where *E* is the single point energy after structure optimization without imaginary frequencies, *E*_total_ is the single point energy of acetamide and *x* (*x* stands for acetamide, EC or DMC), *E*_act_ is the single point energy of acetamide, and *E*_x_ is the single point energy of acetamide, EC or DMC. The dissociation energy was calculated to: Δ*E* = *E*_total_(y) − (*E*_Zn_ + *E*_Cl_ + *nE*_act_), where Δ*E* is the dissociation energy; *E*_total_(y) is the total energy of [ZnCl(acetamide)]^+^, [ZnCl(acetamide)_2_]^+^, and [ZnCl(acetamide)_3_]^+^, respectively; *E*_Zn_, *E*_Cl_, and *E*_act_ are the energy of Zn^2+^, Cl^−^ and acetamide, respectively; *n* is the number of acetamide contained in the Zn(II) complexes. The activation energy was calculated according to the dynamical electron transfer theory using Δ*G* = 0.25 (Δ_r_*G* + *λ*)^2^/*λ*, where Δ*G* is the Gibbs energy of activation, Δ_r_*G* is the standard reaction Gibbs energy for electron transfer process: [ZnCl(acetamide)_n_]^+^  + 2*e*^−^ → Zn + Cl^−^ + n(acetamide), and *λ* is the reorganization energy^23^.

## Results

**Figure 1:**
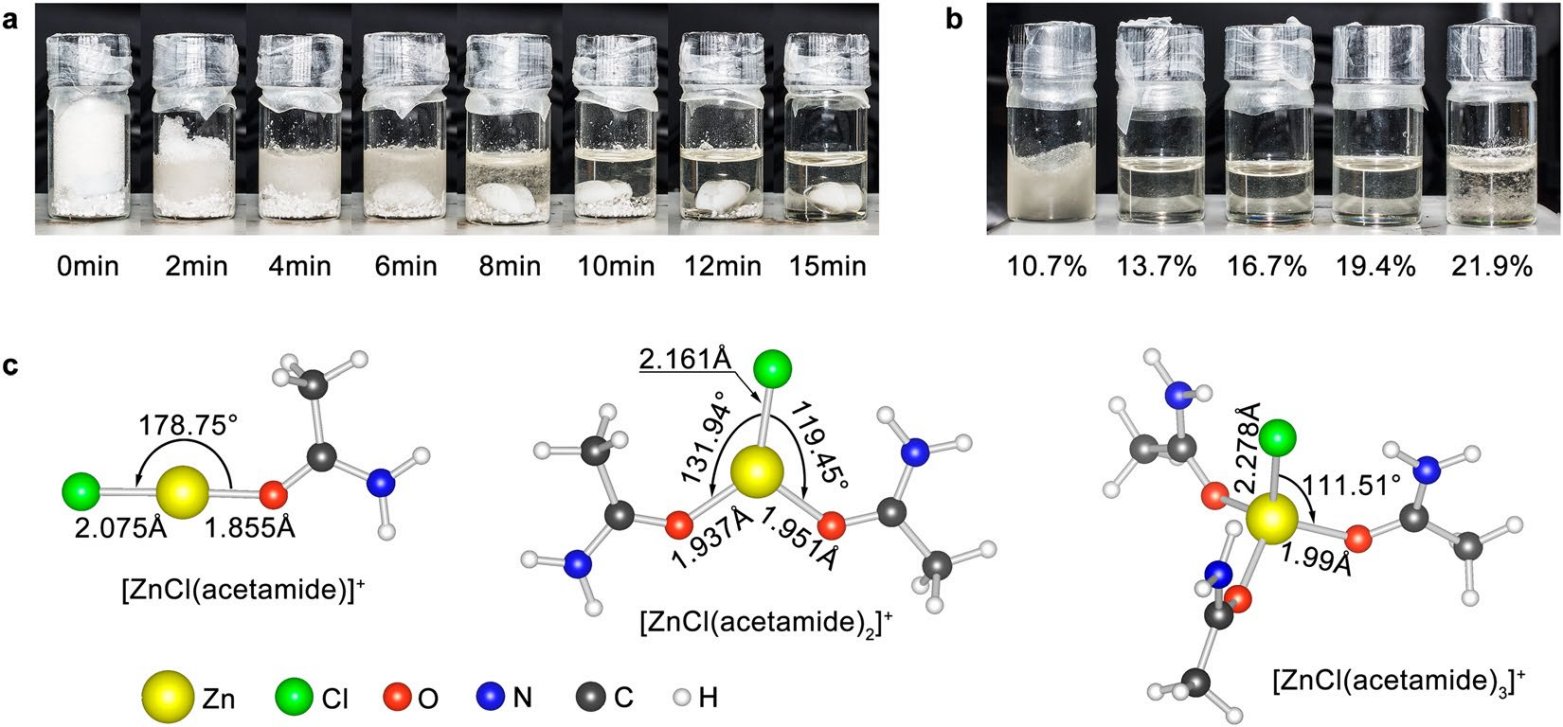
(**a**) Digital images showing the formation process with time of Zn eutectic solvent; (**b**) digital images showing the effect of different ZnCl_2_ molar fraction on the formation of Zn eutectic solvent; (**c**) optimized coordination geometries of Zn(II) complexes in the eutectic solvent.

The Zn-based eutectic solvent was readily prepared by simply mixing ZnCl_2_ with acetamide under constant stirring at 60 °C. The mixing process was quick that a transparent liquid formed within 15 min (Fig. 1a). The lower and upper molar fraction limits of ZnCl_2_ for the formation of a stable eutectic solvent were determined to be 13.7% and 19.4%, respectively (Fig. 1b). The existence of Zn^2+^ in the eutectic solvent have been determined to be [ZnCl(acetamide)]^+^, [ZnCl(acetamide)_2_]^+^ and [ZnCl(acetamide)_3_]^+^ ^24^. Optimized coordination geometry of these Zn(II) complexes (Fig. 1c) revealed that [ZnCl(acetamide)]^+^ was likely to be linear structure, while [ZnCl(acetamide)_2_]^+^ and [ZnCl(acetamide)_3_]^+^ was likely to be triangle and tetrahedral structure. These Zn(II) complexes should not formed through a hydrogen bond, but more probably through an oxygen bond between the acetamide and Zn^2+^ center. Such a coordination geometry suggested that the upper limit of ZnCl_2_ at which the Zn eutectic solvent could be held should depend upon the stability of acetamide molecule. The Zn eutectic solvent would not decompose but rather lose the organic component at elevated temperatures close to the boiling point of the pure component.

**Figure 2:**
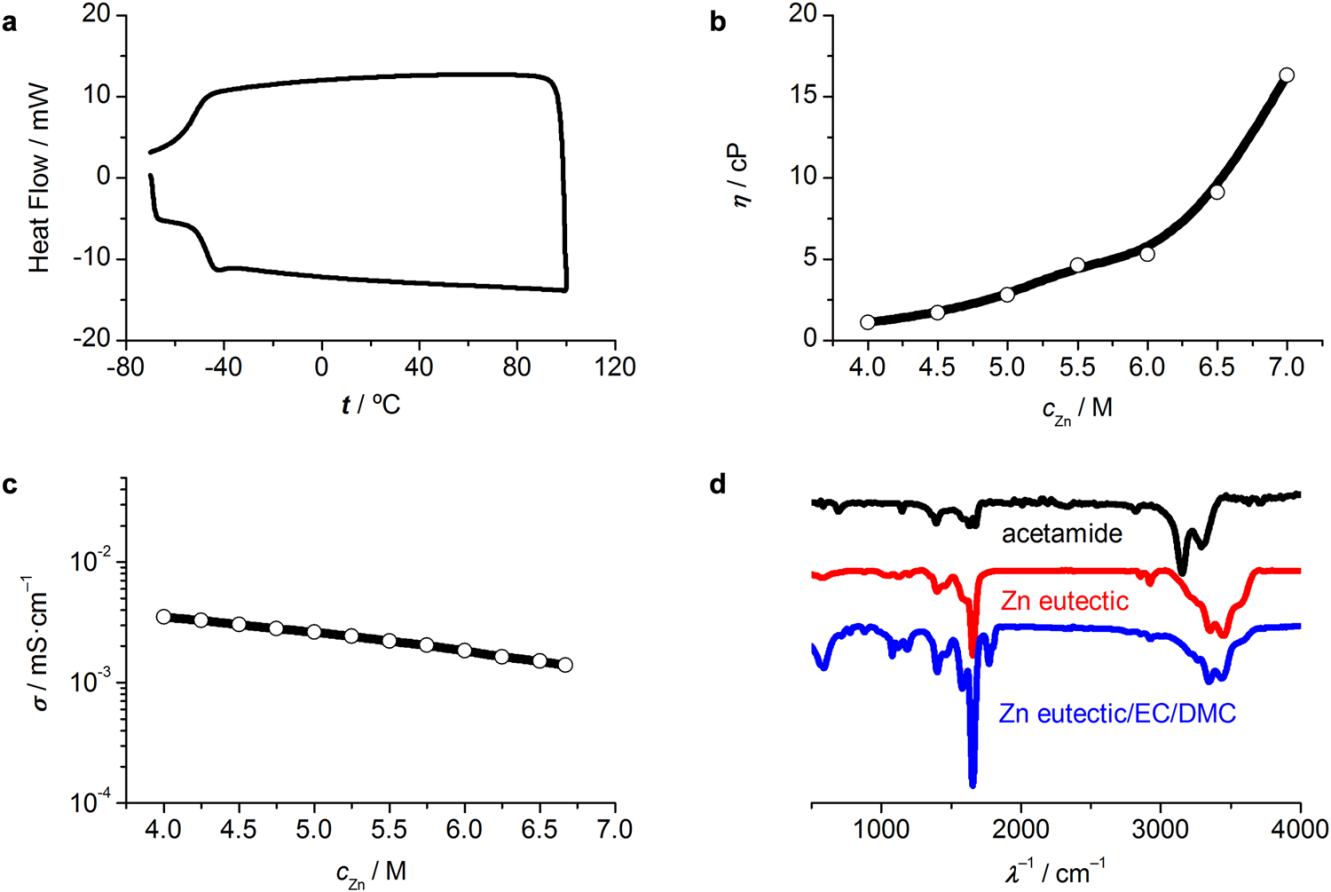
Characterization of Zn eutectic solvent. (**a**) Power-compensated DSC heating and cooling hysteresis profile of the as-prepared Zn eutectic solvent in a temperature range from −80 to 100 °C; (**b**) room-temperature viscosity and (**c**) ionic conductivity of Zn eutectic solvent; (**d**) FTIR spectra of acetamide, Zn eutectic solvent and Zn eutectic mixture. The equivalent concentration of ZnCl_2_ in the Zn eutectic mixture is 5 M.

Power-compensated differential scanning calorimetry (DSC) measurement indicated that the as-prepared Zn eutectic solvent showed a lowest freezing temperature of −38 °C at a ZnCl_2_ molar ratio of 16.7%, and no phase change was observed up to 100 °C (Fig. 2a). Such a wide temperature range should be superior to most aqueous and aprotic electrolyte used for RFBs. However, it’s necessary to point out that, as found in other eutectics^25,26^, the as-prepared Zn eutectic solvent exhibited relatively high viscosity of 10–25 cP in the ZnCl_2_ concentration rage of 4.5–6.5 M (Fig. S2, Supplementary material), which should be resulted from the charge delocalization occurred through the hydrogen bonding between halide ion and hydrogen-donor moiety^25^. Decrease the viscosity would allow sufficient mass transport and thus enhance the conductivity, because the molar conductivity of an electrolyte should be inversely proportional to the viscosity of the solvent according to the Stokes’ law^27^. According to the calculation results, by adding ethylene carbonate/dimethyl carbonate (EC/DMC, 3:7, v- v) into the Zn eutectic solvent, interaction among the solvent molecules could be weakened by means of tuning differing degrees of hydrogen bonding among acetamide, EC and DMC molecules (Fig. S3, Supplementary material). The resultant Zn eutectic mixture showed decreased viscosity of 1–16 cP with equivalent Zn^2+^ concentration ranging from of 4 to 7 M (Fig. 2b) and ionic conductivity of 10^−3^ S·cm^−1^ (Fig. 2c), comparable to the nonaqueous electrolytes for Li-ion batteries^28^. It should be noted that the participation of EC and DMC molecules lead to weakened molecular interactions rather than affected the coordination geometries of Zn^2+^ as evident by Fourier Transform infrared (FTIR) spectra (Fig. 2d). On one hand, the obvious red-shifted broad band between 3600 and 3100 cm^−1^ could be assigned to the hydrogen bond forming between amine and carbonyl groups of acetamide^29^. This broad band showed a redshift after the adding of EC/DMC solvent, indicating that molecular interaction by hydrogen bonding was weakened, consistent with aforementioned calculation results. On the other hand, pure acetamide showed a characteristic band at ca. 1670 cm^−1^ is assigned to the vibration of carbonyl group, which showed a blue shift to ca. 1650 cm^−1^ after the formation of Zn eutectic solvent by resulting from the formation of metal oxygen bond between Zn^2+^ and C=O group of acetamide. This band remained at the same wavenumber after the adding of EC/DMC solvent, suggesting that the introducing of EC and DMC molecules into the Zn eutectic solvent did not affect the coordination geometries of Zn^2+^.

The cell used to evaluate the electrochemical performance of Zn-based eutectic mixture as anolyte was carried out in a home-built cell similar to previous reports^30^. The cell was composed of carbon electrode and Zn foil as current collector in cathode and anode side, respectively, Zn-based eutectic mixture as anolyte, a Li^+^-ion conductive membrane as separator, and other solutions containing redox species with proper redox potential as catholyte (Fig. S4, Supplementary material). In this demonstration, LiI and [Fe(phen)_3_](BF_4_)_2_ (phen = 1,10-phenanthroline) were used as the active material in the catholyte because [Fe(phen)_3_]^3+^/[Fe(phen)_3_]^2+^ redox couple shows good structural stability both in oxidative and reductive states, fast redox kinetics with rate constant on the order of 10^−3^ cm·s^−1^ at room temperature, high redox potential of 0.68 V vs. Ag/AgCl, and stable cyclability as well as cycling efficiencies and in aprotic electrolyte (Figs S5–S7, Supplementary material), while I_3_^−^/I^–^ redox couple shows reliable reversibility as well as high solubility in aqueous electrolyte^31–33^. The as-fabricated cell was in its discharged state. Upon charging, [Fe(phen)_3_]^2+^ was oxidized into [Fe(phen)_3_]^3+^ on the carbon electrode in the cathode chamber, while Zn(II) complex in the eutectic mixture was electroplated onto the Zn foil in the anode chamber. Simultaneously, the charge carrier, Li^+^, diffused from the catholyte into the anolyte to balance the charge.

**Figure 3:**
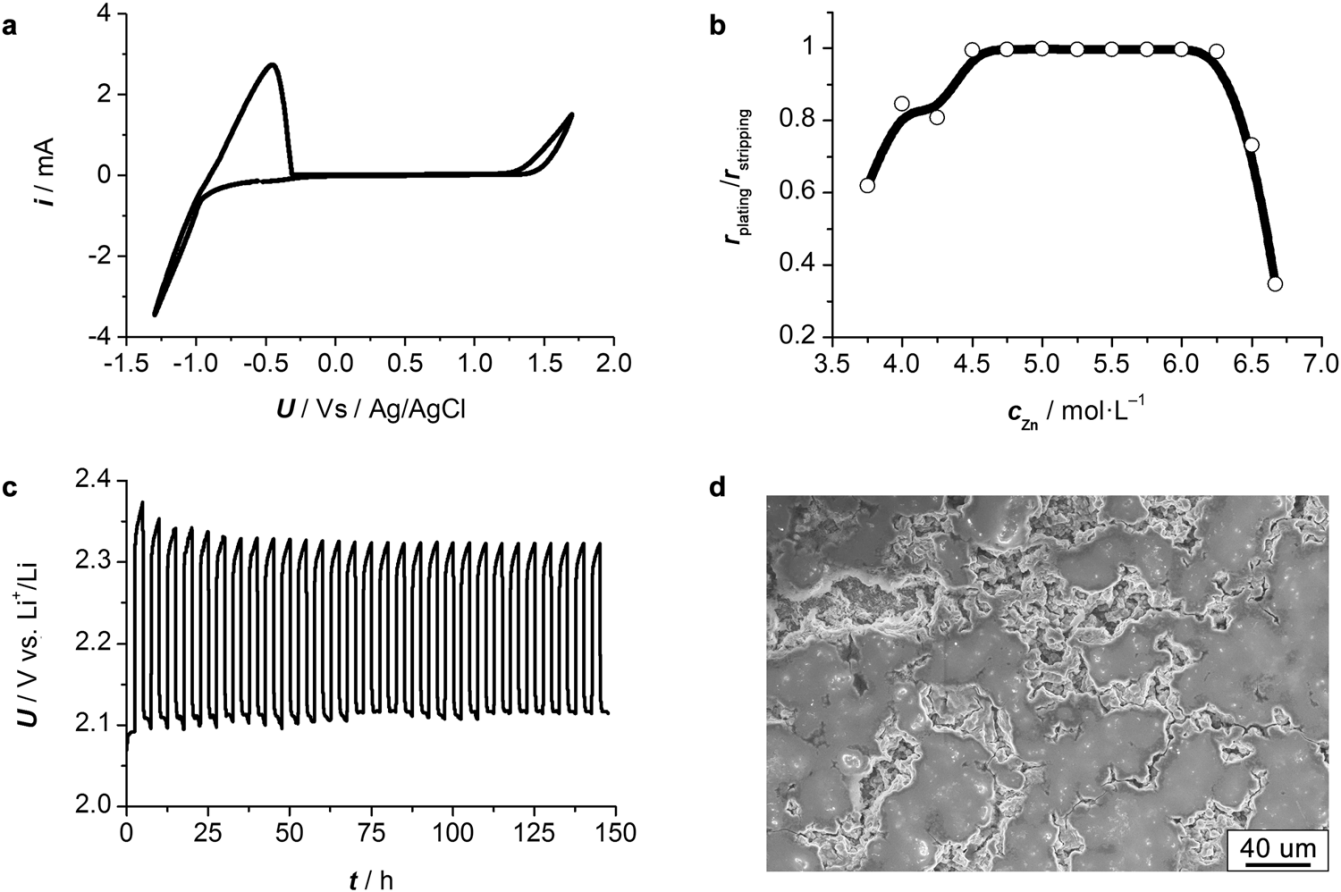
Electrochemical characterizations of 5 M Zn eutectic mixture | separator | Li half-cell. (**a**) Cyclic voltammogram profile recorded at a sweeping rate of 10 mV·s^−1^ showing the electrochemical window of Zn eutectic mixture; (**b**) electroplating/electrostripping ratio (***r***_plating_/***r***_stripping_) versus ZnCl_2_ concentration (*c*_Zn_) of Zn eutectic mixtures at room temperature. The cut-off voltage is set to 0.9–2.3 V vs. Li^+^/Li and the current density is set to 0.2 mA·cm^−2^; (**c**) short-term cycling performance. The charging and discharging time is fixed to 2.5 hrs, and the current density is set to 0.2 mA·cm^−2^; (**d**) SEM image showing the typical morphology of electroplated Zn on Zn foil in a fully discharged half-cell.

The electrochemical properties of Zn eutectic mixture were firstly investigated to address some general concerns about its capability for practical battery application. The electrochemical stability of Zn eutectic mixture was first investigated using a conventional three-electrode configuration with glassy carbon as the working and counter electrodes, and silver chloride electrode as the reference electrode. Cyclic voltammogram profile of the Zn eutectic mixture (Fig. 3a) demonstrated that no obvious current fluctuation was observed in the potential range of −0.8 to 1.3 V vs. Ag/AgCl. Potential lower than −0.8 V vs. Ag/AgCl resulted in Zn^2+^ electroplating, while potential higher than 1.3 V vs. Ag/AgCl led to the decomposition of acetamide. The Zn eutectic mixture showed an impressive electrochemical stable window up to 1.3 V vs. Ag/AgCl, which is comparable with most organic electrolyte used in Li^+^-ion batteries^34^. Moreover, the utilization ratio of Zn eutectic mixture with variable ZnCl_2_ concentration was examined in a half-cell (5 M Zn eutectic mixture | separator | Li). Zn eutectic mixture showed a high electroplating/electrostripping ratio close to 100% when the concentration of ZnCl_2_ between 4.5 and 6.25 M, compared with that obtained when the concentration of ZnCl_2_ below 4.5 or above 6.25 M (Fig. 3b). This phenomenon could be attributed to phase separation of Zn eutectic mixture according to the general phase diagram of eutectics^26^. Furthermore, short term cycling test (Fig. 3c) revealed that the half-cell showed stable charge/discharge potential around 2.33/2.12 V vs. Li^+^/Li. The potential gap was ca. 0.25 V at the first a few cycles but gradually decreased to ca. 0.2 V in the subsequent cycles, which should be attributed to decreased internal resistance owing to the enhanced wettability between Zn eutectic mixture and Zn foil, as well as improved permeation of Li^+^-ion through the separator. It’s important to note that Zn deposited from deep-eutectic-solvents tends to have a compact microcrystalline structure, in contrast to Zn deposited from aqueous electrolytes which tends to have a dendritic structure in the absence of strong base additives^16^. The current collector after electroplating for sufficient time (Fig. S8, Supplementary material) was examined by *Ex-situ* SEM, which confirmed a dendrite-free Zn electroplating (Figs 3d and S9, Supplementary material), possibly resulting from the lowered electroplating exchange current due to acetamide coordination with Zn^2+^ ^35^.

**Figure 4:**
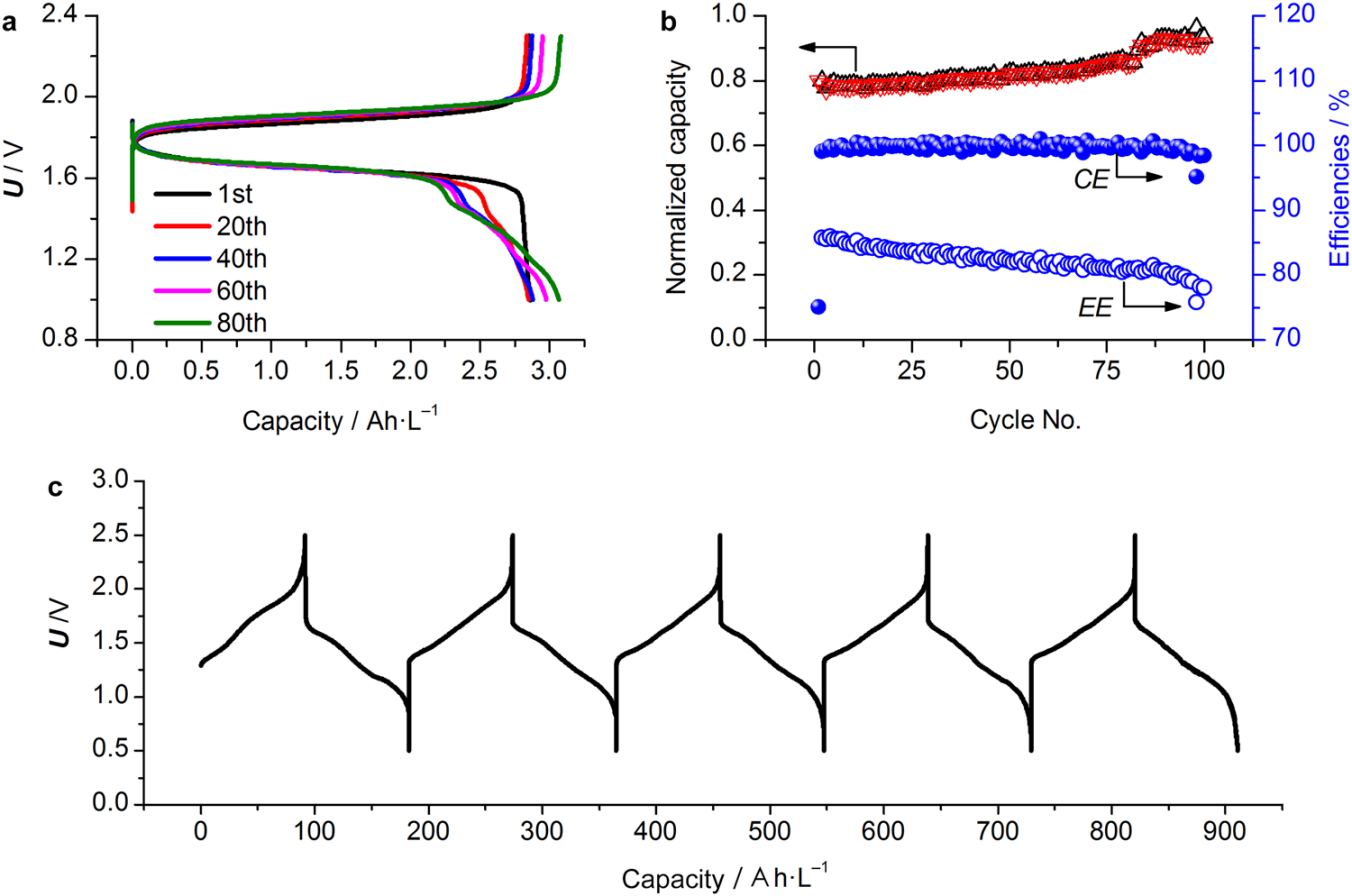
Electrochemical characterizations of Zn eutectic mixture | [Fe(phen)_3_](BF_4_)_2_ and Zn eutectic mixture | LiI cells. (**a**) Charge/discharge profiles of Zn eutectic mixture | [Fe(phen)_3_](BF_4_)_2_ cell assembled from 5 M Zinc eutectic mixture as anolyte and 0.2 M [Fe(phen)_3_](BF_4_)_2_ and 0.8 M LiTFSI in EC/DMC as catholyte. The volume ratio of the anolyte and catholyte is 1:2; (**b**) corresponding charge/discharge capacity, Coulombic efficiency (CE) and energy efficiency (EE). The normalized capacity stands for the value of the actual capacity divided by the maximum capacity under such a cell configuration; (**c**) charge/discharge profiles of Zn eutectic mixture | LiI cell assembled using 6.25 M Zinc eutectic mixture as anolyte and 3 M LiI aqueous solution as catholyte with a volume ratio of 1:2. The capacity is calculated based on the volume of the Zinc eutectic mixture.

In the following full cell test, [Fe(phen)_3_]^3+^/[Fe(phen)_3_]^2+^ redox couple dissolved in EC/DMC with 0.8 M LiTFSI was used (5 M Zn eutectic mixture | separator | 0.2 M [Fe(phen)_3_]^3+^/[Fe(phen)_3_]^2+^). The cell showed a pair of anodic and cathodic peaks centering at 2.1 and 1.2 V, respectively (Fig. S10, Supplementary material). Galvanostatic cycling profiles of the full cell demonstrated stable cycling performance (Fig. 4a). During discharge, the voltage decreased monotonically until reaching the 1 V cutoff. While upon charging, the voltage jumped to 1.8 V, and then increased monotonically until reaching the 2.4 V cutoff. The average overpotentials were similar during the discharge and recharge processes, suggesting that the electrochemical steps involved in the forward and reverse cell reactions exhibited similar activation energies. The Coulombic and energy efficiency maintained ca. 100% and 78%, respectively (Fig. 4b). During the first 30 cycles, the cell showed a steady increase in capacity, which eventually stabilized to ca. 3 A h·L^−1^ based on the total volume of the anolyte and catholyte. To further demonstrate the capability of the Zn eutectic mixture, excess concentrated LiI aqueous solution was used as the catholyte (5 M Zn eutectic mixture | separator | 3 M LiI). The cell was able to deliver a volumetric capacity of ca. 90 A h·L^−1^ based on the volume of the anolyte (Fig. 4c), corresponding to a utilizable concentration ca. 1.7 M out of 6.25 M ZnCl_2_ in the Zn eutectic mixture could be reversibly used. The Coulombic efficiency of the full cell was nearly 100% in the initial cycle, which was in consistent with that achieved in the half cell with ZnCl_2_ concentration between 4.5 M and 6.25 M. In consideration of two-electron redox reaction of Zn^2+^, such a high concentration exceeded that of the commonly used stability limit (<1.7 M) for RFBs^36^.

## Discussion

**Figure 5:**
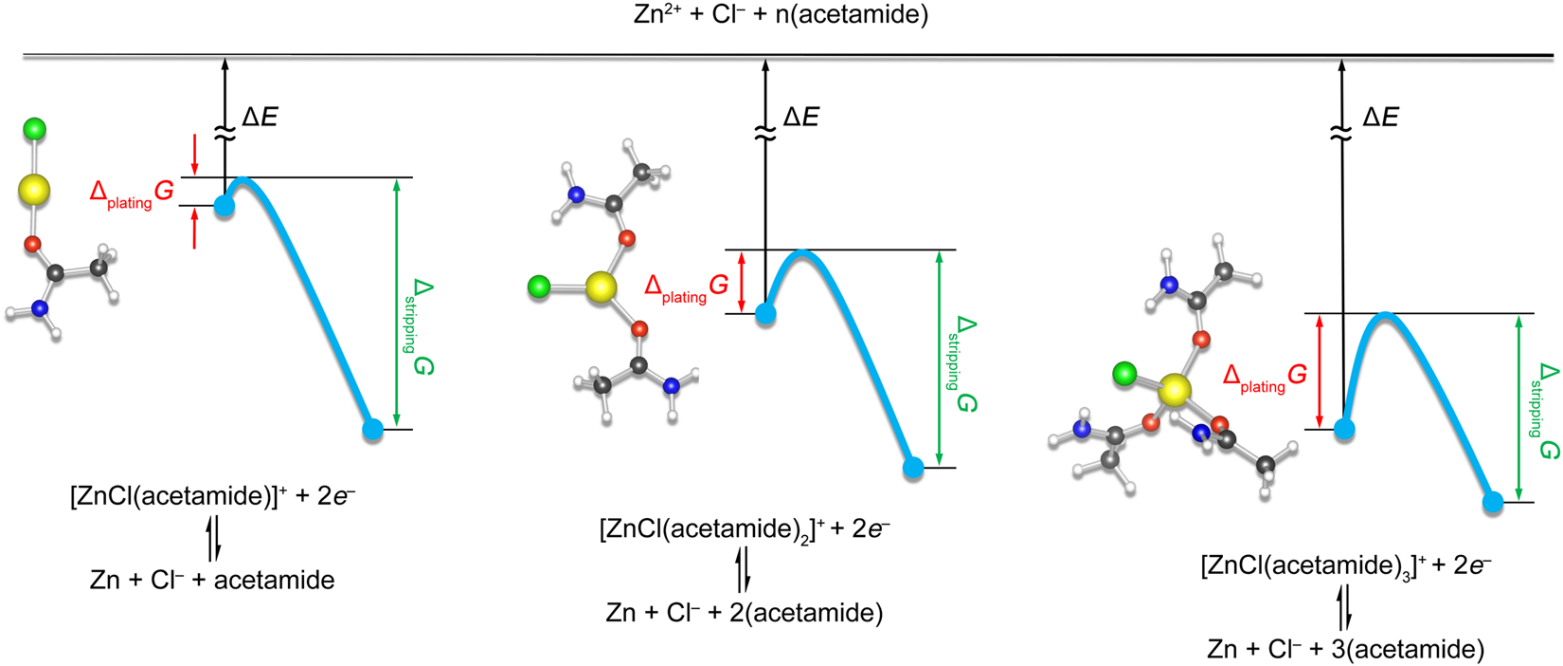
Schematic demonstration of the dissociation energy (Δ*E*) of Zn(II) complexes and activation energy of [ZnCl(acetamide)]^+^, [ZnCl(acetamide)_2_]^+^ and [ZnCl(acetamide)_3_]^+^ during electroplating (Δ_plating_*G*) and electrostripping (Δ_stripping_*G*) processes in the Zn eutectic mixture.

The mechanism of Zn electroplating/electrostripping in the Zn eutectic mixture was studied using DFT-based computational simulation (Fig. 5). The calculation results (Table S1, Supplementary material) indicated that the dissociation energy to break the coordination geometry of [ZnCl(acetamide)]^+^, [ZnCl(acetamide)_2_]^+^ and [ZnCl(acetamide)_3_]^+^ was −498.88, −555.65 and −602.08 kcal·mol^−1^, respectively, indicating that, on one hand, the strength of coordinated interaction followed the order of [ZnCl(acetamide)_3_]^+^ > [ZnCl(acetamide)_2_]^+^ > [ZnCl(acetamide)]^+^ with [ZnCl(acetamide)_3_]^+^ being the most stable geometry; on the other hand, Zn electroplating/electrostripping processes were not likely to occur through direct dissociation of Zn(II) complexes in the Zn eutectic mixture due to such a high dissociation energy. Further analysis on the activation energy of the three coordination geometries revealed that [ZnCl(acetamide)]^+^ and [ZnCl(acetamide)_3_]^+^ were responsible for Zn electroplating and electrostripping, respectively. In the electroplating process, [ZnCl(acetamide)]^+^ showed an activation energy of 0.12 kcal·mol^−1^, compared with those of 9.08 kcal·mol^−1^ and 13.54 kcal·mol^−1^ for [ZnCl(acetamide)_2_]^+^ and [ZnCl(acetamide)_3_]^+^, respectively, indicating that the electroplating process should be more thermodynamically preferable for [ZnCl(acetamide)]^+^ being electrochemically reduced to Zn than the other two geometries. The Zn electroplating processes were supposed to composed of two dynamic equilibrium processes and one major path through reactions (1–3):

1 $$[\mathrm{ZnCl}{({\rm{acetamide}})}_{3}]{\rm{Cl}}\leftrightarrow [{\rm{ZnCl}}{({\rm{acetamide}})}_{2}]{\rm{Cl}}+{\rm{acetamide}}$$

2 $$[\mathrm{ZnCl}{({\rm{acetamide}})}_{2}]{\rm{Cl}}\leftrightarrow [{\rm{ZnCl}}({\rm{acetamide}})]{\rm{Cl}}+{\rm{acetamide}}$$

3 $$2[\mathrm{ZnCl}({\rm{acetamide}})]{\rm{Cl}}+2{e}^{-}\leftrightarrow {\rm{Zn}}+[{\rm{ZnCl}}{({\rm{acetamide}})}_{2}]{\rm{Cl}}+{{\rm{Cl}}}^{-}$$

While in the electrostripping process, however, the activation energy of [ZnCl(acetamide)_3_]^+^ was found to be 148.063 kcal·mol^−1^, lower than those of the other two geometries of 188.483 and 163.536 kcal·mol^−1^ for [ZnCl(acetamide)]^+^ and [ZnCl(acetamide)_2_]^+^, respectively. Therefore, it’s likely Zn electrostripping occurred preferably following one major path via reaction (4):

4 $${\rm{Zn}}+{{\rm{Cl}}}^{-}+3{\rm{acetamide}}-2{e}^{-}\leftrightarrow {[{\rm{ZnCl}}{({\rm{acetamide}})}_{3}]}^{+}$$

## Conclusions

we present here a successful demonstration of the utilization of Zn-based deep-eutectic-solvent that can be readily prepared using inexpensive and abundant materials as anolyte for hybrid RFBs. The new chemistry applied here is inherently environmental-friendly and elementally abundant. The Zn eutectic mixture is capable to utilize 1.7 M electroactive Zn^2+^ thus delivers a volumetric capacity ca. 90 A h·L^−1^ with good cycling efficiencies. Zn electroplating/electrostripping in this anolyte shows good reversibility and no sign of dendrite formation. Zn electroplating/electrostripping process is investigated by DFT-based computational simulation, based on which the Zn redox reaction mechanism has been proposed. The cell architecture is compatible with traditional hybrid RFBs and can be readily scale-up. Together with its low cost and environmental benignity, such an anolyte may find its application in stationary electric energy storage with the rapidly growing quest for greener and more sustainable batteries.

## Electronic supplementary material

Supplementary data
