# Supplementary material for: Zn-based eutectic mixture as anolyte for hybrid redox flow batteries
Source: Sci Rep. 2018 Apr 10;8:5740. doi: 10.1038/s41598-018-24059-x (PMC5893549; doi:10.1038/s41598-018-24059-x)
Supplement: Supplementary file 1 — Supplementary data [file 41598_2018_24059_MOESM1_ESM.docx]

Supplementary data for:

**Zn-based eutectic mixture as liquid anode for hybrid redox flow batteries**

Yiyu Wang, ^a,†^ Zhihui Niu, ^a,†^ Qi Zheng, ^a^ Changkun Zhang, ^a^ Jing Ye, ^b^ Gaole Dai, ^a^ Yu Zhao, ^a,*^ Xiaohong Zhang ^a,*^

^a^ Institute of Functional Nano & Soft Materials (FUNSOM), Jiangsu Key Laboratory for Carbon-Based Functional Materials & Devices, Soochow University, 199 Renai Road, Suzhou Industrial Park, Suzhou, Jiangsu 215123, P. R. China.

^b^ Testing & Analysis Centre, Soochow University, 199 Renai Road, Suzhou Industrial Park, Suzhou, Jiangsu 215123, P. R. China.

^†^ These authors contribute equally to this work

Corresponding Author

Email: yuzhao@suda.edu.cn (Y. Zhao), xiaohong_zhang@suda.edu.cn (X. Zhang)


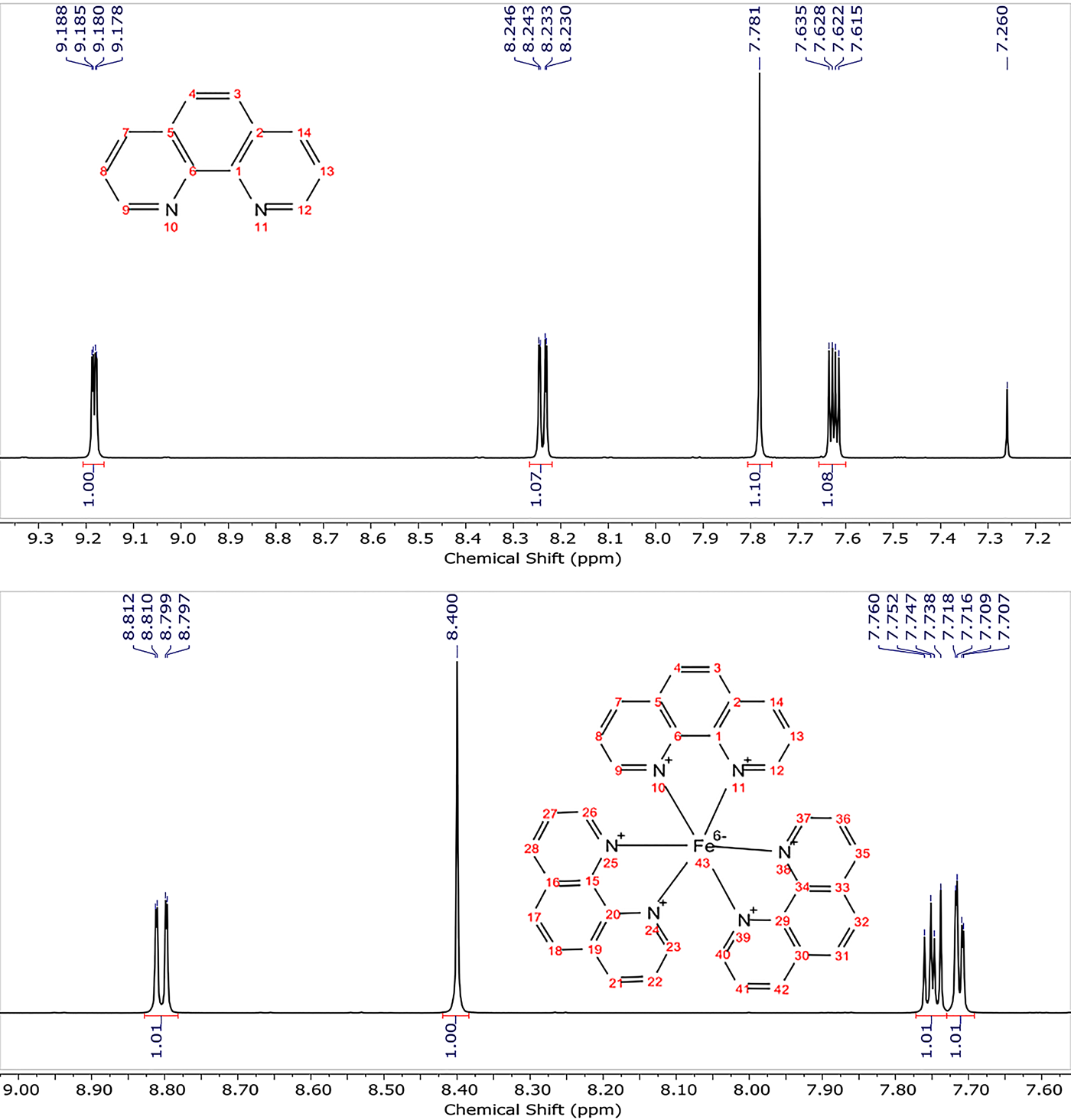


**Fig. S1.** ^1^H NMR of 1,10-phenanthroline and the as-prepared [Fe(phen)_3_](BF_4_)_2_.


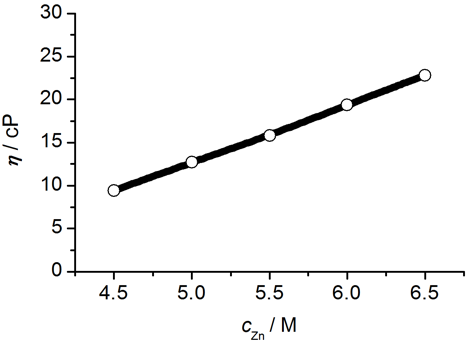


**Fig. S2.** Viscosity of Zn eutectic solvent at different ZnCl_2_ concentration in the eutectic solvent.


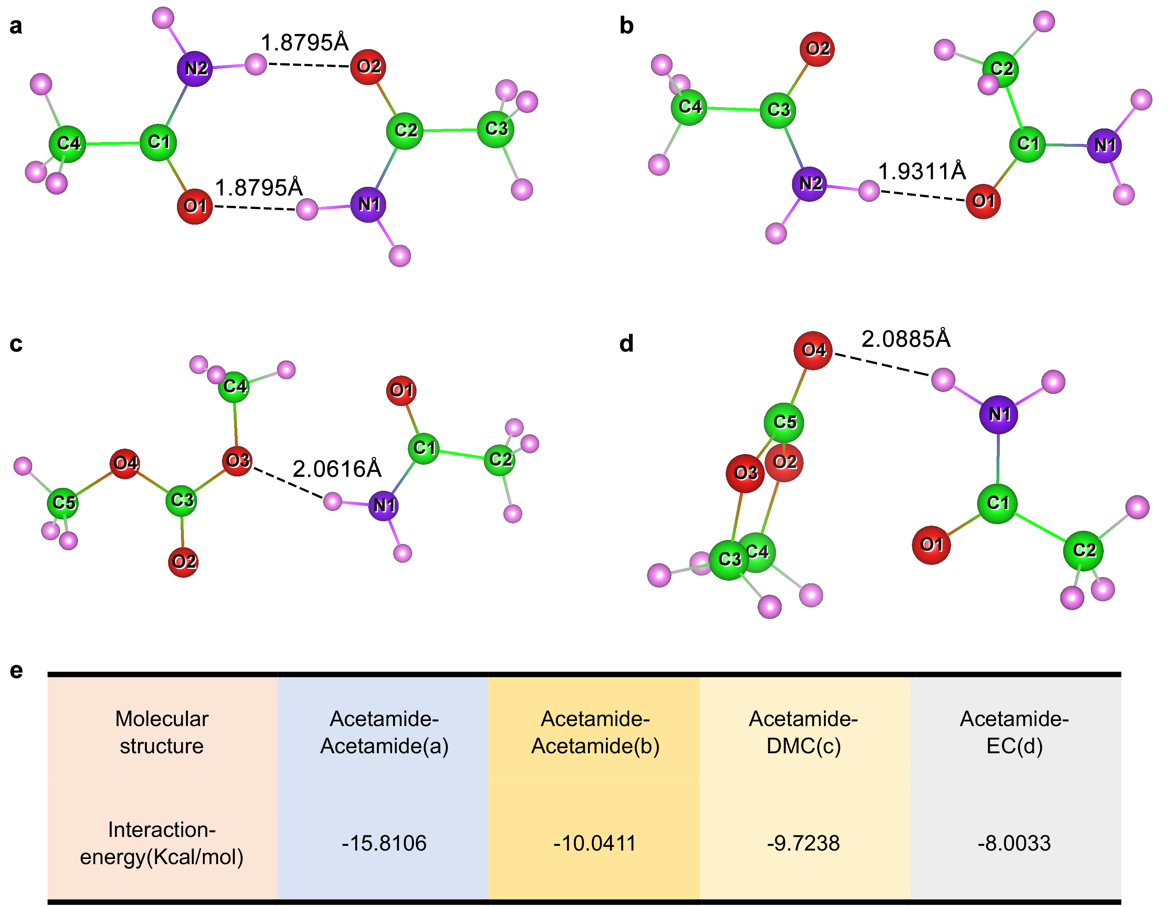


**Fig. S3.** Optimized configurations of hydrogen bond interaction of acetamide-acetamide interaction via two hydrogen bonds (a), acetamide-acetamide interaction via one hydrogen bond (b), acetamide-DMC interaction (c), and acetamide-EC interaction (d). (e) corresponding hydrogen bond interaction energy. The hydrogen bonding interaction energy of the four molecular configurations demonstrates that the interaction energy follows acetamide-acetamide (two hydrogen bonds) > acetamide-acetamide (one hydrogen bond) > acetamide-DMC > acetamide-EC. Therefore, adding EC/DMC to the Zn eutectic solvents can decrease the viscosity effectively mainly due to weakening or breaking the intra N-H···O hydrogen bonds of acetamide-acetamide through the generation of new intermolecular hydrogen bonds of acetamide-DMC and acetamide-EC.


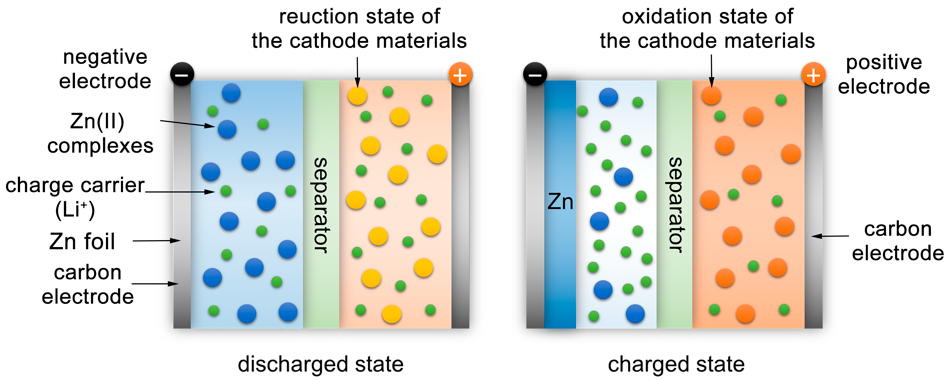


**Fig. S4.** Schematic demonstration of the cell structure and composition in its fully discharged and charged states. A NASICON-type Li_1+x+3z_Al_x_(Ti,Ge)_2−x_Si_3z_P_3−z_O_12_ Li^+^-ion conducting membrane (LICGC^®^ AG-01, Ohara Corp.) with Li^+^-ion conductivity of 10^−4^ S·cm^−1^ at room temperature is served as the separator. The geometric surface area of the separator is 0.5 mm^2^, and the thickness of the anode and cathode is 1 mm and 2 mm, respectively.


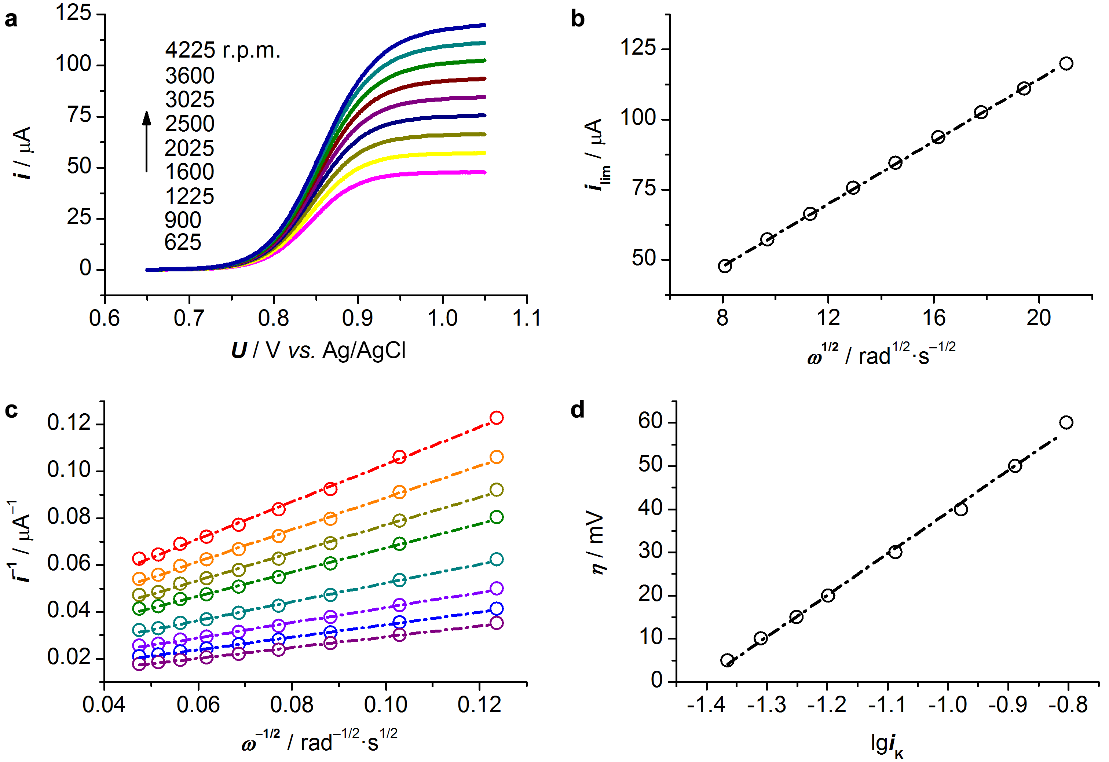


**Fig. S5.** RDE investigation of [Fe(phen)_3_]^3+^/[Fe(phen)_3_]^2+^ redox couple. (a) RDE voltammograms of [Fe(phen)_3_](BF_4_)_2_ electrolyte; (b) corresponding limiting current (*i*) *vs.* square root of rotation speed (*ω*^1/2^); (c) corresponding kinetic current (*i*_K_) as a function of *ω*^−1/2^ upon [Fe(phen)_3_]^2+^ oxidation at different overpotentials (*η*); (d) *η* as a function of lg*i*_K_ upon [Fe(phen)_3_]^2+^ oxidation. The *x*-intercept gives the lg of the exchange current (*i*_0_). The *i* vs. *ω*^1/2^ shown in (b) is fitted with a straight line, with the slope defined by the Levich equation as *i* = 0.620×*nFD*^2/3^*Aω*^1/2^*ν*^–1/6^*C*, where *n*=1, *F*=96,485 C·mol^–1^, electrode area *A*=7.07 mm^2^, kinematic viscosity of the electrolyte *ν* ≈ 3.2×10^–2^ cm^2^·s^–1^, and bulk concentration of [Fe(phen)_3_]^2+^ *C* = 2.5×10^–6^ mol·cm^–3^. This gives *D* values of 5.2×10^–6^ cm^2^·s^–1^. The reciprocal of the current at *η* of 5, 10, 15, 20, 30, 40, 50 and 60mV is plotted versus the reciprocal of *ω*^–1/2^ as shown in (c). The *i*^–1^ for each *η* is fitted with a straight line as shown in (d); the intercept gives the reciprocal of *i*_K_, the current in the absence of mass transport limitations (the extrapolation to infinite rotation rate). A plot of lg*i*_K_ versus *η* is linearly fitted. According to the Butler–Volmer equation, *i*_0_ = *nFAk*_0_*C*, the exchange current (*i*_0_) is given by the *x*-intercept in the lg*i*_K_–*η* profile, from which *k*_0_ is calculated to be ca. 2.3×10^–3^ cm·s^–1^.


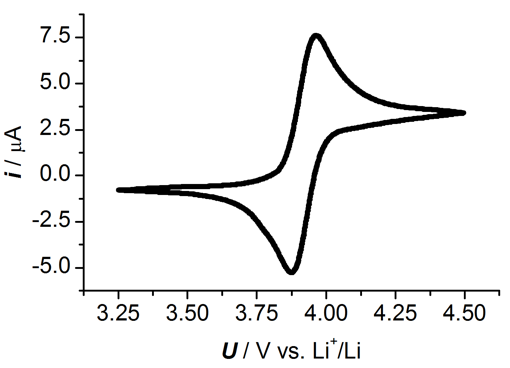


**Fig. S6.** CV profile of [Fe(phen)_3_]^3+^/[Fe(phen)_3_]^2+^ redox couple. The electrolyte uses EC/DMC (3:7, v:v) as solvent, LiTFSI (250 mM) as supporting electrolyte, and [Fe(phen)_3_](BF_4_)_2_ (10 mM).


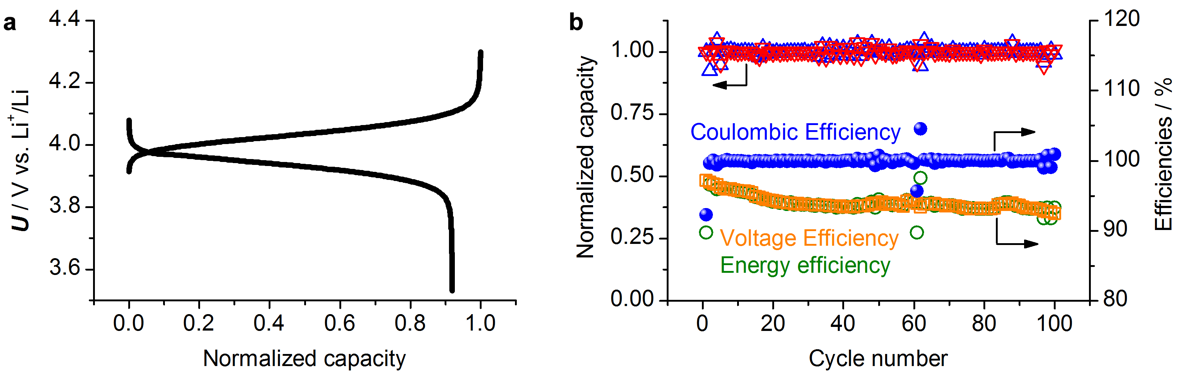


**Fig. S7.** (a) Initial charge/discharge profiles and (b) normalized capacity retention and corresponding Coulombic and energy/voltage efficiencies of 0.2 M [Fe(phen)_3_](BF_4_)_2_ | separator | Li half-cell. The half-cell shows stable capacity retention without obvious capacity loss during the measured 100 cycles. The corresponding Coulombic and energy/voltage efficiencies are approximately 99.5% and 94%/94% respectively. The electrolyte used in the half-cell is 0.2 M [Fe(phen)_3_](BF_4_)_2_ and 0.8 M LiTFSI in EC/DMC (3:7, v:v). The normalized capacity stands for the value of the actual capacity divided by the maximum capacity could be achieved in a 1mm-thick, 0.5 cm^2^ electrode (0.268 mA h).


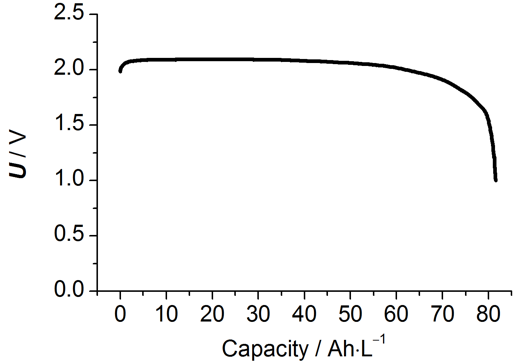


**Fig. S8.** Zn electroplating from 6.25 M Zn eutectic mixture | separator | Li half-cell upon full discharge. The electrolytes of the half-cell are composed of 100 μL Zn eutectic mixture in a 1mm-thick, 0.5 cm^2^ electrode. Zn eutectic mixture delivers a specific capacity of ca. 82 A h·L^–1^, limited by available Zn^2+^ concentration in the Zn eutectic mixture.


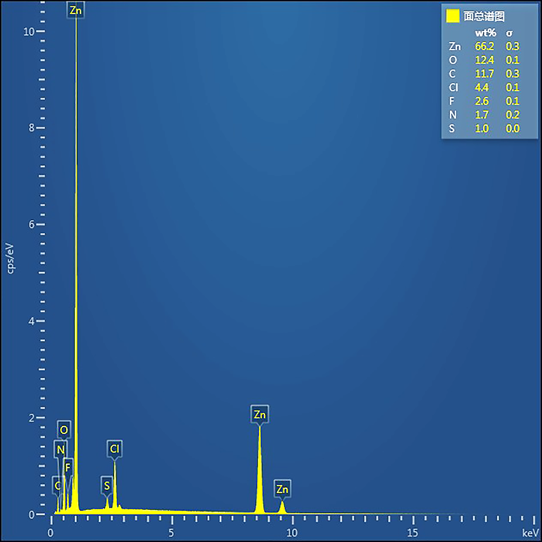


**Fig. S9.** Ex-situ surface energy dispersive X-ray analysis suggesting Zn is successfully electroplated from 5 M Zn eutectic mixture | separator | Li half-cell upon full discharge. The signals of O, C, Cl, F, N and S are from traces of residual acetamide and LiTFSI that have not been removed by washing, and surface oxide layers of electroplated Zn as a result of exposure to air and moisture.


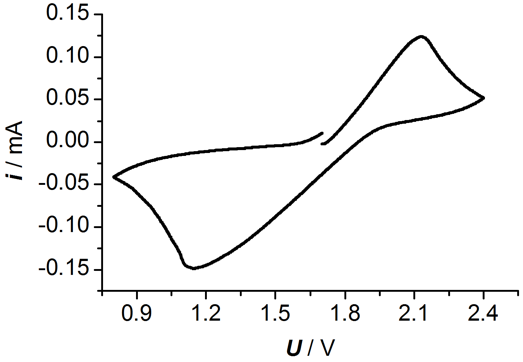


**Fig. S10.** CV profile of 5 M Zn eutectic mixture | separator | 0.2 M [Fe(phen)_3_]^3+^/[Fe(phen)_3_]^2+^ full-cell at a sweeping rate of 0.1 mV·s^–1^. The CV profile is measured in a conventional two-electrode cell with Zn foil serves as both the counter and reference electrode.

**Table S1.** Calculated dissociation energy (Δ*E*) and activation energy (Δ*G*) of Zn electroplating and electrostripping of three coordinated geometries of Zn^2+^ in Zn eutectic mixture.

|  | **[ZnCl(Acetamide)]^+^** | **[ZnCl(Acetamide)_2_]^+^** | **[ZnCl(Acetamide)_3_]^+^** |
| --- | --- | --- | --- |
| ***E*_initial_ (a.u.)** | –2448.785 | –2658.146 | –2867.490 |
| ***E*_final_ (a.u.)** | –2449.086 | –2658.392 | –2867.705 |
| **Δ*E* (kcal·mol^–1^)** | –498.88 | –555.65 | –602.08 |
| **Δ_plating_*G* (kcal·mol^–1^)** | 0.116 | 9.083 | 13.539 |
| **Δ_stripping_*G* (kcal·mol^–1^)** | 188.483 | 163.536 | 148.063 |
| **Reaction heat (kcal·mol^–1^)** | –188.367 | –154.453 | –134.524 |
